# Comparative Analysis of Therapeutic Efficacy and Adverse Reactions among Various Thrombolytic Agents

**DOI:** 10.3390/toxics12070458

**Published:** 2024-06-25

**Authors:** Chenxi Xie, Naying Zheng, Mingmei Li, Zhiyang Zhang, Dongqin Huang, Meizhu Xiao, Dongdong Chen, Chengyong He, Zhenghong Zuo, Xintan Chen

**Affiliations:** 1 Chest Pain Center, Anxi County Hospital, Quanzhou 362300, China; kudistar@163.com (C.X.); 15259794157@163.com (M.L.); 15106084165@163.com (Z.Z.); huangdongqin610@163.com (D.H.); 18960200260@163.com (M.X.); chendongdong244@163.com (D.C.); 2 State Key Laboratory of Cellular Stress Biology, School of Life Sciences, Xiamen University, Xiamen 361005, China; 18750249346@163.com (N.Z.); hecy@xmu.edu.cn (C.H.)

**Keywords:** thrombolytic drug, thrombus, bleeding, thrombin

## Abstract

Thrombosis is a major health concern that contributes to the development of several cardiovascular diseases and a significant number of fatalities worldwide. While stent surgery is the current recommended treatment according to the guidelines, percutaneous coronary intervention (PCI) is the optimal approach for acute myocardial infarction (AMI). However, in remote areas with limited resources, PCI procedures may not be feasible, leading to a delay in treatment and irreversible outcomes. In such cases, preoperative thrombolysis becomes the primary choice for managing AMI in remote settings. The market for thrombolytic drugs is continuously evolving, and identifying a safe and effective thrombolytic agent for treating AMI is crucial. This study evaluated Urokinase, Alteplase, and Recombinant Human TNK Tissue-type Plasminogen Activator for Injection (rhTNK) as representatives of first-, second-, and third-generation thrombolytic drugs, respectively. The research included in vitro thrombolysis experiments, exposure of human cardiomyocytes, zebrafish tail vein injections, and vascular endothelial transgenic zebrafish models. The findings revealed that rhTNK is the most effective thrombolytic drug with the least adverse effects and lowest bleeding rate, highlighting its potential as the preferred treatment option for AMI. The order of thrombolytic effectiveness was Urokinase < Alteplase < rhTNK, with adverse effects on cardiomyocytes post-thrombolytic therapy ranking similarly as Urokinase < Alteplase < rhTNK, while the bleeding rate after thrombolysis followed the order of Urokinase > Alteplase > rhTNK.

## 1. Introduction

Acute myocardial infarction (AMI) is marked by myocardial necrosis stemming from acute and prolonged ischemia and hypoxia within the coronary arteries [1]. This condition affects nearly 3 million individuals globally, with over 1 million deaths attributed to it annually in the United States alone [2]. In 2019, cardiovascular diseases resulted in 18.6 million deaths globally. Noteworthy is that cardiovascular disease fatalities comprised 23% to 35% of all deaths during this timeframe [3]. Ischemic heart disease (47%) and stroke (40%) were accountable for the majority (87%) of cardiovascular disease deaths in Asia [4]. Given Asia’s vast population size, diverse ethnicities, cultures, socioeconomic conditions, and healthcare systems, the region encounters multiple obstacles concerning cardiovascular disease prevention and management.

AMI necessitates prompt reperfusion therapy to enhance the clinical outcomes. As per the established clinical guidelines, percutaneous coronary intervention (PCI) stands out as the preferred initial approach for reperfusing AMI [5,6]. However, access to hospitals capable of performing timely PCI procedures is limited. AMI is defined by myocardial necrosis induced by acute and persistent ischemia and hypoxia within the coronary arteries. Clinically, the typical symptoms of AMI include severe and lasting retrosternal pain (pain behind the breastbone), which may radiate to the left arm, neck, jaw, or back. The pain is usually not relieved by rest or nitrates, which are medications that dilate the blood vessels and increase the blood flow to the heart. This is often accompanied by elevated serum myocardial enzyme levels, progressive electrocardiogram alterations, and potential complications such as arrhythmias, shock, and heart failure, all of which pose life-threatening risks. In situations where patients who are located in remote areas or when medical constraints prevent immediate PCI, thrombolysis emerges as the primary treatment choice for acute myocardial infarction [7]. Previous research has provided evidence that administering thrombolysis before PCI in patients with AMI can significantly reduce the total duration of ischemia and improve the success rate of vascular revascularization in affected individuals [8]. This study analyzed tabulated data from 22 randomized trials involving at least 100 patients each (totaling 50,246 patients) that compared fibrinolytic therapy with a placebo or control. These trials were reported between 1983 and 1993 and focused on evaluating the relationship between treatment delay and short-term mortality (up to 35 days). The findings of the study reveal that the beneficial effect of fibrinolytic therapy is significantly greater in patients who presented within 2 h after symptom onset compared to those who sought treatment at a later time [8].

Thrombolytic drugs have undergone iteration and updating in recent years, with current pre-operatively used agents including Streptokinase, Alteplase (tPA), Reteplase (rPA), and Tenecteplase (TNK-tPA) [9]. Among these, Streptokinase exhibits a moderate treatment effect but is associated with a higher risk of adverse reactions such as bleeding, allergic responses, and fever [10]. Alteplase, rPA, and TNK-tPA demonstrate effective thrombolytic outcomes, albeit with a potential for causing hemorrhage, particularly intracranial hemorrhage in patients [11,12,13]. The primary concern with thrombolytic drugs is their propensity to induce significant bleeding in patients during the process of clot dissolution to reopen blood vessels. Consequently, the search for a thrombolytic drug with potent thrombolytic effects and a low bleeding rate is paramount for the clinical treatment of AMI patients.

In this study, we aimed to compare the thrombolytic effects and side effects of different thrombolytic drugs. Three widely used, clinically and commercially available thrombolytic drugs in clinical practice—Urokinase, Alteplase, and rhTNK were selected. We prepared 24 h and 48 h in vitro thrombi using citric acid plasma, followed by the addition of these three thrombolytic drugs to assess their respective thrombolytic effects. Furthermore, AC16 cells were exposed to clinical doses of these thrombolytic agents. To simulate the in vivo effects of these drugs, zebrafish were utilized as a model, with injections of Urokinase and Alteplase through the tail vein and observing the bleeding rates and vascular endothelial damage in the zebrafish. Overall, our study holds significance in providing valuable insights into the selection of thrombolytic drugs for clinical application, emphasizing the importance of considering both the thrombolytic efficacy and potential side effects during drug selection.

## 2. Materials and Methods

### 2.1. In Vitro Thrombus Preparation

According to previous reports, we prepared the human thrombus in vitro [14,15]. Blood was collected from blood vessels pre-treated with an anticoagulant solution of adenine citrate phosphate dextrose (ACPD). This collected blood was promptly transported to the laboratory and stored at room temperature, with all clots obtained within 5 h. To create the thrombi, 1.5 mL centrifuge tubes were utilized as molds, with 0.9 mL of blood added to each tube, followed by the addition of 1 mL of 0.06% CaCl_2_. The tubes were incubated in a 37 °C water bath for about 5 h before being extracted and then stored at 4 °C. Thrombus formation typically initiates within the initial 48 h. Based on this timeframe, we prepared 24 and 48 h thrombi at 4 °C. To enhance coagulation on the thrombus surface, a brief 5 s centrifugation step was performed. Subsequently, the supernatant serum was discarded, leaving behind the thrombus, and the size and photographs of the thrombus were recorded.

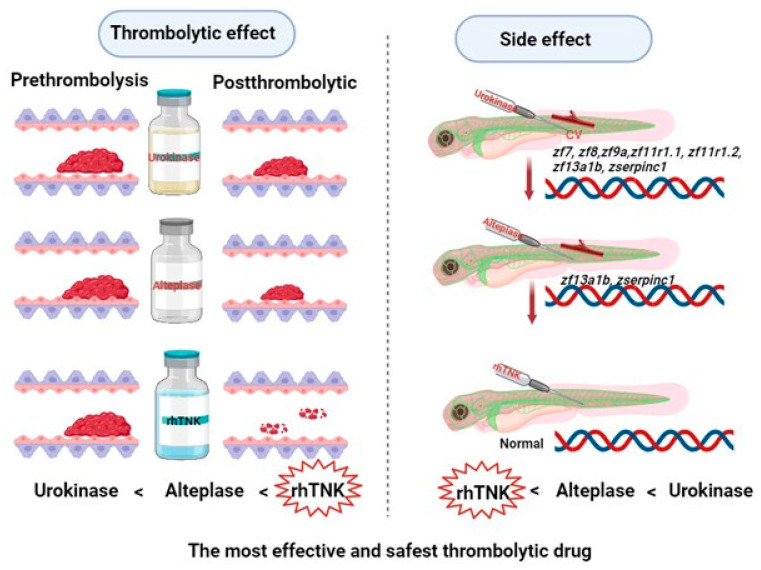



### 2.2. Extracorporeal Thrombolysis

To assess the effectiveness of different thrombolytic drugs, formulations were prepared following the manufacturer’s instructions. Based on the clinical dosages of these three thrombolytic drugs, formulations were created as follows: 2500 U/mL Urokinase, 0.2 mg/mL Alteplase, and 0.032 mg/mL rhTNK. The thrombolytic test was conducted in a water bath at 37 °C and lasted for 2 h. This experimental setup allowed for the evaluation of the thrombolytic efficacy of each drug formulation under standardized conditions.

### 2.3. Cell Culture

The culture medium for AC16 cells was prepared by adding 10% Fetal Bovine Serum (FBS) (Biological Industries, Beit Haemek, Israel) to DMEM/F12 (1:1) medium from Gibco, Grand Island, NY, USA. The AC16 cells were then cultured at 37 °C in a 5% CO_2_ atmosphere. When the cell confluence reached 80–90%, 0.25% trypsin-EDTA from TransGen Biotech, Beijing, China, was utilized to detach the cells for further processing.

### 2.4. Cell Viability

We plated 1 × 10^4^ cells per well in 96-well plates and incubated them overnight at 37 °C in a 5% CO_2_ atmosphere. Subsequently, the cells were exposed to the three thrombolytic drugs for 24 h and 48 h, respectively. The cell viability was assessed using an Methylthiazolyldiphenyl-tetrazolium bromide (MTT) assay (*n* = 6), and the absorbance at 490 nm was measured using a microplate reader (Bio-Rad, Hercules, CA, USA) to quantify the cell viability.

### 2.5. Immunofluorescence

Based on previous reports, we conducted immunofluorescence assays [16]. Specifically, we seeded 3 × 10^5^ cells per well in 6-well plates and cultured them overnight at 37 °C in a 5% CO_2_ atmosphere. Subsequently, the cells were exposed to the three thrombolytic drugs for 24 and 48 h, respectively. Following drug exposure, the cells were rinsed three times with 1× phosphate buffer saline (PBS), fixed with 4% paraformaldehyde (PFA) for 20 min, and then stained with the primary antibodies β-TUBULIN (Zen Bioscience, Chengdu, China) antibodies (1:200) at 4 °C overnight. The next day, the cells were rewarmed at 37 °C for 30 min, stained with the secondary antibody, Alexa Fluor 488 (Proteintech, Chicago, IL, USA) solution (1:400), and incubated at 37 °C for one hour in the dark. Finally, the nuclei were stained with 4′,6-diamidino-2-phenylindole (DAPI) (1:10,000) for 3 min, and images were captured using an LSM 900+Airyscans confocal microscope (Zeiss, Oberkochen, Germany).

### 2.6. Zebrafish Culture

The wild-type TU zebrafish and Tg (*fli1*:EGFP) zebrafish were cultured in accordance with the guidelines set by the Animal Ethics Committee of Xiamen University. The zebrafish were maintained under a circadian rhythm of 14 h light and 10 h dark, with the culture medium temperature maintained at 28 ± 1 °C. The zebrafish culture medium contained 7–8 mg/L of dissolved oxygen, and the water pH was maintained at 7.0–7.4. Imaging and observation were conducted after injecting the tail veins of 72 h post-fertilization (hpf) zebrafish larvae with the three thrombolytic drugs, as previously reported [17]. Each thrombolytic drug was injected into six replicates, with each replicate consisting of 30 larvae, while the control group was injected with normal saline (0.9% NaCl solution).

### 2.7. Real-Time Quantitative PCR (qPCR)

In the study, twenty larvae from each replicate were pooled into a subsample for analysis. The mRNA expression levels were measured using qPCR following previously established methods [18]. The total RNA was extracted from whole larvae using TRIzol reagent (TaKaRa, Tokyo, Japan) following the manufacturer’s protocol. The first-strand cDNA was synthesized from 500 ng of the total RNA using the SurperMix Kit (AG, Bingjie, China). Subsequently, qPCR analysis was performed using the SYBR Green qPCR reagent kit (Abclone, Wuhan, China) on the Mx3000P Real-Time PCR system (Agilent Technologies, Santa Clara, CA, USA) according to the manufacturer’s instructions. To normalize the expression levels of *f7*, *f8*, *f9a*, *f11r1.1*, *f11r1.2*, *f13a1b*, *serpinc1*, *f5*, *f9b*, and *f13a1.1*, the expression level of *β-actin* was used as a reference gene. The data were analyzed using the 2^−ΔΔCt^ method. The primers used for the PCR are listed in Table S1.

## 3. Results

### 3.1. In Vitro Thrombus Preparation

**Figure 1:**
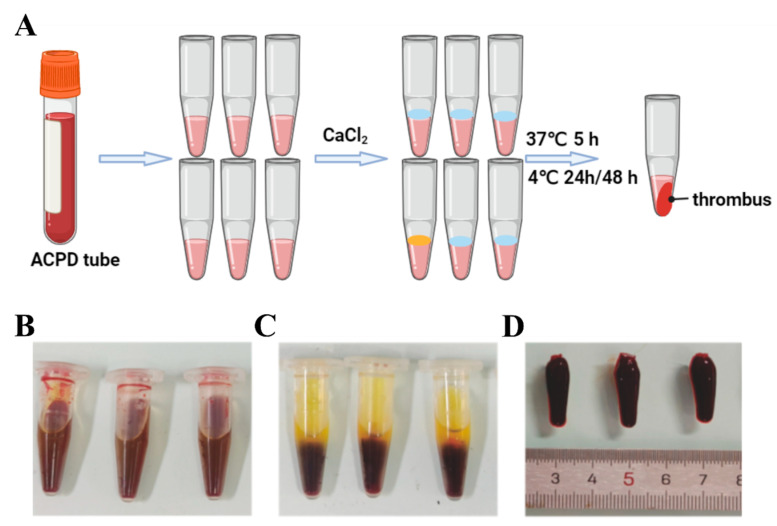
In vitro thrombosis preparation. (**A**) Diagram of thrombus preparation model in vitro, (**B**) diagram of thrombus preparation process in vitro, (**C**) thrombus precipitation, (**D**) thrombosis.

The thrombus preparation process involved the use of an ACPD tube (Figure 1A). Initially, 1 mL of blood preserved in the ACPD tube was transferred into a centrifuge tube (Figure 1B). Subsequently, CaCl_2_ was introduced to initiate thrombus coagulation (Figure 1C), leading to the formation of the thrombus which was then obtained by removing the supernatant (Figure 1D).

### 3.2. Comparison of the Effects of Different Thrombolytic Drugs on 24-Hour and 48-Hour Thrombi

**Figure 2:**
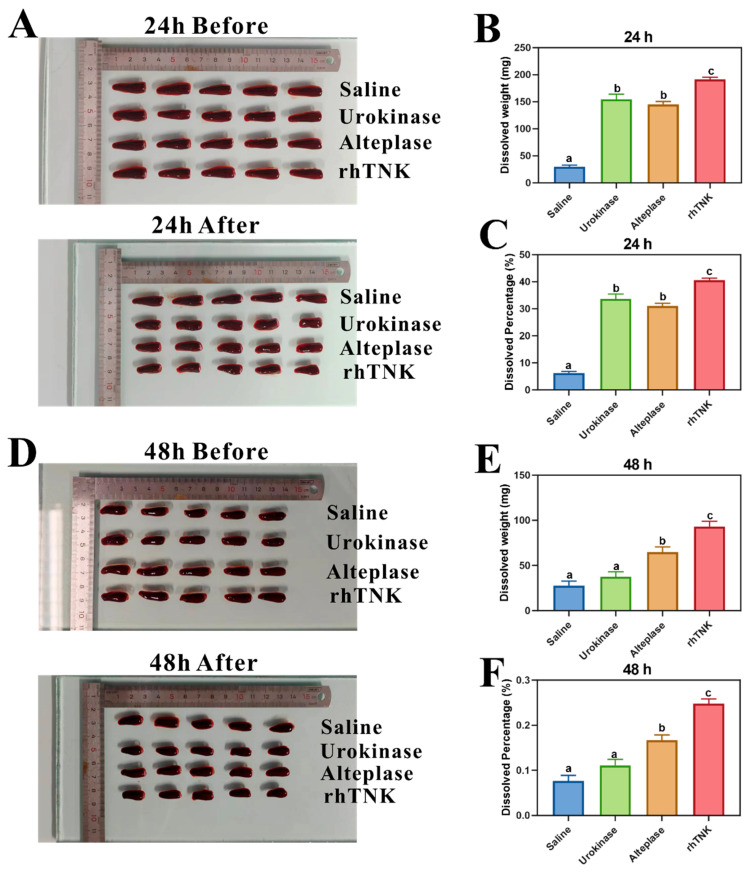
Comparison of thrombolytic effect of different thrombolytic drugs. (**A**) Typical images before and after thrombolytic therapy of 24 h thrombus, (**B**) dissolved weight of different thrombolytic drugs’ treatment of 24 h thrombus, (**C**) dissolved percentage of different thrombolytic drugs’ treatment of 24 h thrombus, (**D**) typical images before and after thrombolytic therapy of 48 h thrombus, (**E**) dissolved weight of different thrombolytic drugs’ treatment of 48 h thrombus, (**F**) dissolved percentage of different thrombolytic drugs’ treatment of 48 h thrombus. The data are presented as the mean ± SE. The different letters on the bar indicate the significant change.

Based on the evolution of thrombolytic drugs and their widespread clinical application, we selected representative thrombolytic drugs from the first, second, and third generations for comparative analysis [19,20,21,22,23]. Specifically, Urokinase [24], Alteplase [25] and rhTNK [22] were chosen as representatives. As shown in Figure 2, these three thrombolytic agents exhibited efficacy in thrombus dissolution at both the 24 h and 48 h marks. For the 24 h thrombus, the thrombus dissolution weight in the control group was 29.94 ± 5.88 mg. The Urokinase and Alteplase treatment groups demonstrated similar dissolution weights (154.74 ± 18.59 mg and 145.1 ± 10.92 mg, respectively), with rhTNK exhibiting the highest dissolution weight at 191.4 ± 7.74 mg (Figure 2A–C). The thrombolysis percentage in the 24 h thrombus achieved with rhTNK was the highest among the available agents (Figure 2C). In the case of the 48 h thrombus, Urokinase displayed a modest thrombolytic effect, nearly resembling the control group with minimal thrombolysis observed. The dissolution weight in the control group decreased by 27.86 ± 17.05 mg, while that in the Urokinase group decreased by 37.70 ± 11.99 mg, showing no statistically significant difference. Alteplase exhibited a substantial increase in thrombolytic weight, reaching 64.74 ± 13.07 mg. Conversely, rhTNK showed consistent efficacy compared to the 24 h thrombosis, surpassing the previous generations of thrombolytic drugs with a dissolution weight of 93.10 ± 12.92 mg (Figure 2D–F).

### 3.3. Effects of Different Thrombolytic Drugs on AC16 Cells

**Figure 3:**
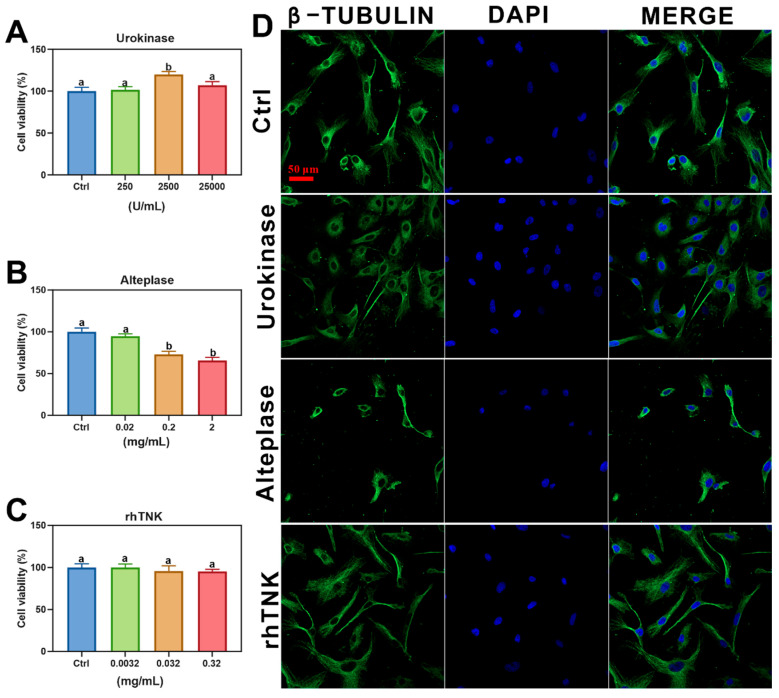
AC16 cell comparing the safety of different thrombolytic drugs. The cell viability of AC16 cells after treatment by Urokinase (**A**), Alteplase (**B**), and rhTNK (**C**). Cytoskeleton stained by β-TUBULIN and nuclear stained DAPI of AC 16 cells after the treatment of the three drugs; green: cytoskeleton, blue: nuclear (**D**). The data are presented as the mean ± SE. The different letters on the bar indicate the significant change.

The effects of three thrombolytic drugs on the cell viability and morphology of human cardiomyocytes (AC16) were observed in our study. The MTT results revealed that 2500 U/mL of Urokinase increased the cell viability from 100.00% ± 11.27 to 119.93% ± 8.96, while 25,000 U/mL of Urokinase did not affect the cell viability (Figure 3A). In contrast, the treatment with Alteplase led to a dose-dependent decrease in the cell viability, with 0.2 and 2 mg/mL of Alteplase reducing the cell viability to 73.08% ± 8.75 and 65.64% ± 9.38, respectively (Figure 3B). Notably, the treatment with rhTNK, representing the third-generation thrombolytic drug, did not alter the viability of the AC16 cells (Figure 3C). Furthermore, the AC16 cells were treated with the three thrombolytic drugs for 2 h, followed by staining with β-TUBULIN to observe the effects on the cytoskeleton. As depicted in Figure 3D, the Urokinase and Alteplase groups, particularly the Alteplase group, exhibited a decreased cell cytoskeleton density. In contrast, the rhTNK treatment did not change the cell cytoskeleton (Figure 3D). To investigate whether residual amounts of these thrombolytic drugs cause myocardial cell damage in vivo, the cytoskeleton and nucleus of the AC16 cells were observed after 48 h of treatment with the three thrombolytic drugs, respectively. The results indicated that Urokinase and Alteplase not only reduced the cell cytoskeleton density but also induced nucleus enlargement in the AC16 cells (Supplementary Figure S1A). Conversely, the cytoskeleton and nuclear morphology of the AC16 cells treated with rhTNK were consistent with those of the cells treated with normal saline (0.9% NaCl solution) (Supplementary Figure S1A).

### 3.4. Effects of the Three Thrombolytic Drugs on the Blood Vessels of Zebrafish

**Figure 4:**
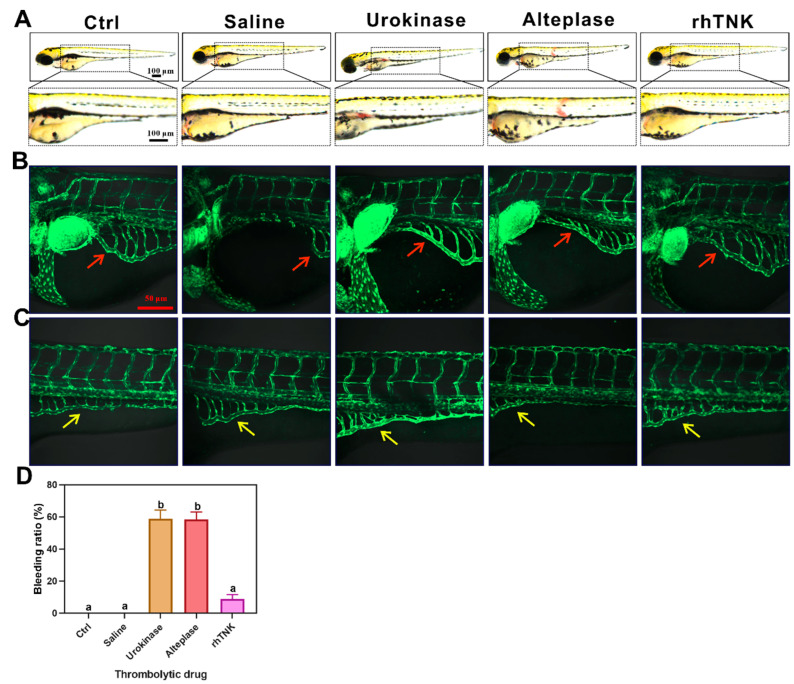
Effects of three thrombolytic drugs on blood vessels of zebrafish. (**A**) Three thrombolytic drugs’ injection induced zebrafish bleeding. Effects of the three thrombolytic drugs on vascular wall and vascular endothelial cells of Tg(*fli1*:EGFP) zebrafish, vessels in the head (**B**), vessels in the tail (**C**). The bleeding ratio after the injection of the three thrombolytic drugs (**D**). The data are presented as the mean ± SE. The different letters on the bar indicate the significant change.

Previous reports have indicated that the main side effect of thrombolytic drugs is the risk of causing bleeding. To further explore the safety profile of the three thrombolytic drugs, we utilized zebrafish as a model to observe the potential bleeding effects following the tail vein injection of these drugs for two hours. The results demonstrated that Urokinase and Alteplase induced bleeding in zebrafish, while there was no observed bleeding in the saline (0.9% NaCl solution) and recombinant tissue-type plasminogen activator (rhTNK) injection groups (Figure 4A). The bleeding rates in the Urokinase and Alteplase injection groups were 58.89% ± 13.28 and 58.33% ± 11.50, respectively. In contrast, the bleeding rate in the rhTNK injection group was significantly lower at 8.89% ± 6.89 compared to the Urokinase and Alteplase groups (Figure 4D). Furthermore, we investigated the effects of these three thrombolytic drugs on blood vessels using Tg(*fli1*:EGFP) transgenic zebrafish, which specifically label vascular endothelial cells. Our observations revealed that Urokinase and Alteplase led to a concentration of cells along the inner wall of blood vessels and an enhancement of the fluorescence signal (Figure 4B,C).

### 3.5. Impact of Three Thrombolytic Drug Injections on the Gene Expression Levels Associated with the Coagulation Pathway

The hemostasis coagulation mechanism is a complex process that encompasses a cascade of coagulation factors [26]. The intrinsic pathway involves factors I (fibrinogen), II (prothrombin), IX (Christmas factor), X (Stuart–Prower factor), XI (plasma thromboplastin), and XII (Hageman factor) [27,28]. The extrinsic pathway involves factors I, II, VII (stability factor), and X. These factors circulate in the bloodstream as zymogens and undergo activation as serine proteases, facilitating the conversion of fibrinogen [29]. Factors II, VII, IX, X, XI, and XII are classified as serine proteases, whereas factors V, VIII, and XIII do not possess serine protease activity [30] (Supplementary Figure S2). 

**Figure 5:**
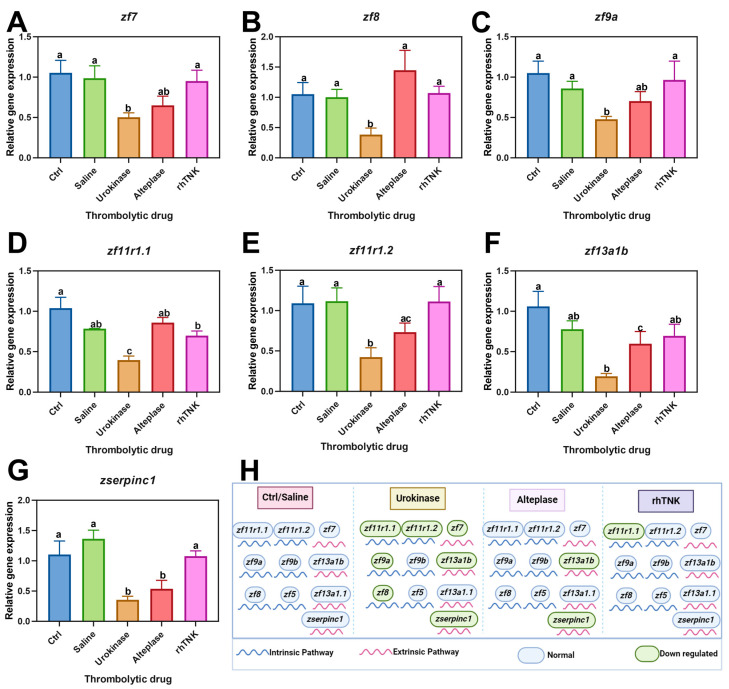
Effect of three thrombolytic drugs on coagulation pathway. The relative expression level of clotting factor was changed by three thrombolytic drugs, *zf7* (**A**), *zf8* (**B**), *zf9a* (**C**), *zf11r1.1* (**D**), *zf11r1.2* (**E**), *zf13a1b* (**F**). The relative expression level of coagulation inhibitor *serpinc1* after the injection of the three thrombolytic drugs (**G**). (**H**) Diagram of mechanism of thrombolytic drugs. The data are presented as the mean ± SE. The different letters on the bar indicate the significant change.

In this study, it was observed that Urokinase significantly down-regulated the expression of serine protease coagulation factors *zf7* by 0.53-fold (Figure 5A), *zf9a* by 0.48-fold (Figure 5C), *zf11r1.1* by 0.40-fold (Figure 5D), and *zf11r1.2* by 0.42-fold (Figure 5E). However, the injection of Alteplase and rhTNK did not significantly alter the expression levels of these serine proteases. Furthermore, the Urokinase injection also led to a significant reduction in the expression levels of non-serine proteases, with *zf8* down-regulated by 0.38-fold (Figure 5B) and *zf13a1b* by 0.19-fold (Figure 5F). Alteplase decreased the expression level of *zf13a1b* by 0.60-fold (Figure 5F), while the expression level of *zf8* remained unchanged (Figure 5B). The expression levels of the non-serine proteases were unaffected by rhTNK (Figure 5B, Supplementary Figure S3A,C).

Moreover, the expression of serpin peptidase inhibitor, clade C (antithrombin), member 1 (*zserpinc1*) was significantly down-regulated by Urokinase by 0.36-fold and Alteplase by 0.54-fold injections but remained unchanged following rhTNK injection (Figure 5G). The expression levels of another serine protease factor, *zf9b*, and two non-serine protease factors, *zf5* and *zf13a1.1*, were not affected by the administration of these three thrombolytic drugs (Supplementary Figure S3B,C).

## 4. Discussion

Thrombosis is a complex process involving platelet activation, fibrin formation, the aggregation of platelets, neutrophil extracellular traps (NETs), and red blood cells (RBCs) to form clots [31]. Recent studies indicate that intravascular cell-derived protein disulfide isomerase PDI plays a significant role in thrombosis, inflammation, and thromboinflammation [32]. The mechanical attributes of a blood clot play a pivotal role in its durability and ability to withstand dissolution. These attributes are dictated by the structural features at multiple tiers, encompassing molecular, individual fiber, and branched fiber network levels [33,34]. This structural complexity contributes to the resilience of blood clots and their ability to withstand forces that could disrupt them. In addressing the challenges posed by thrombus-related diseases, thrombolytic therapy has become a widely utilized approach to promote clot dissolution. Thrombolytic agents work by initiating the fibrinolytic pathway, which involves catalyzing the conversion of plasminogen to plasmin and accelerating the degradation of fibrin. By promoting fibrin breakdown, thrombolytic therapy aims to restore normal blood flow, alleviate vessel occlusion, and reduce the adverse effects associated with thrombosis [35,36]. Overall, understanding the intricate processes involved in thrombosis and the mechanisms of thrombolytic therapy is essential for developing effective treatment strategies for thrombus-related disorders and improving patient outcomes.

The in vitro thrombo-growth model described involves creating a “growth clot” using citric plasma, calcium chloride, and thrombin, followed by the addition of new citric plasma. The excess calcium chloride present in the citric plasma slowly releases, leading to coagulation around the plasma and causing the growth of the clot. This model has been utilized to assess the anticoagulant efficacy of direct thrombin inhibitors, demonstrating that clot growth is inhibited in a concentration-dependent manner. As such, this clot growth model serves as a valuable primary test for evaluating the compounds with anticoagulant properties in laboratory settings. Calcium ions (Ca^2+^) are pivotal in the clotting cascade, and sodium citrate, a prevalent anticoagulant in the blood collection, functions by chelating calcium ions. Consequently, citrate plasma solely necessitates the addition of calcium ions to reestablish its clotting capacity. The fibrin clot formed in this model contains a high concentration of calcium ions, which are released from the plasma, leading to coagulation and clot growth [37,38]. In this model, the weight of the fibrin clot increases in a concentration-dependent manner with calcium chloride. The impact of a direct thrombin inhibitor, such as agatroban, on clot growth can be assessed by directly adding it to the system to observe if the clot growth is halted. Overall, this in vitro thrombo-growth model provides a means to simulate thrombus formation in vivo and offers a simple yet effective tool for evaluating thrombolytic drugs and studying their effects on clot formation and dissolution.

The in vitro thrombolytic experiments conducted revealed the comparative effects of Urokinase, Alteplase, and rhTNK on the dissolution of 24 h and 48 h thrombi. The results indicated that Urokinase and Alteplase had similar effects on the dissolution of the 24 h thrombus, while rhTNK exhibited significantly higher thrombolytic effects in both scenarios. Specifically, for the more challenging 48 h thrombus, the order of thrombolysis effectiveness was rhTNK > Alteplase > Urokinase, highlighting the superior thrombolytic ability of rhTNK to break down clots over an extended period. These findings are consistent with previous reports indicating that rhTNK is a genetically engineered variant of rt-PA (recombinant tissue plasminogen activator) with improved fibrin specificity [39]. Furthermore, a phase II trial of rhTNK-tpa demonstrated a significant increase in myocardial infarction thrombolysis (TIMI) levels 2–3 compared to rt-PA, underscoring the enhanced efficacy of rhTNK as a third-generation thrombolytic agent [22]. These results suggest that rhTNK holds promise as an effective thrombolytic drug with significant potential for clinical applications in treating thrombotic disorders. The experiment on the thrombolytic effect of different concentrations of rhTNK revealed that even at low concentrations, rhTNK demonstrated thrombolytic effects. This was observed to be true even for older thrombi, specifically those aged 48 h, where rhTNK displayed a notable thrombolytic effect. These findings suggest that rhTNK exhibits thrombolytic activity across a range of concentrations, making it a promising candidate for the dissolution of older thrombi and offering potential clinical benefits in treating thrombotic conditions.

The human cardiomyocyte experiments conducted revealed the distinct effects of different thrombolytic agents on the cell viability and structural integrity. The administration of Urokinase led to an increase in the cell viability of human cardiomyocytes. This aligns with previous research demonstrating the promotion of corneal epithelial migration and nerve regeneration by Urokinase [40]. In contrast, treatment with Alteplase resulted in a decrease in cell viability, suggesting potential harm to cardiomyocytes. Additionally, both Urokinase and Alteplase treatments led to a reduction in myocardial cytoskeleton fibers, indicating structural damage to myocardial cells. This implies that the use of these two thrombolytic drugs in cardiac settings may carry a risk of heart damage. On the other hand, the cell viability, cytoskeleton structure, and cell morphology observed in the rhTNK treatment group closely resembled those in the control group. This suggests that rhTNK treatment did not adversely affect the cell viability or structural integrity of human cardiomyocytes. These results indicate that rhTNK may be a safer option with no apparent side effects on the heart based on this experiment’s findings. In summary, these observations highlight the potential differences in the impact of various thrombolytic agents on cardiomyocyte health, with rhTNK showing promise as a safer alternative in terms of preserving the cell viability and structural integrity of human cardiomyocytes.

Previous studies have indicated that the injections of Urokinase and Alteplase lead to bleeding [20,25,41,42,43,44]. Consistent with this, the zebrafish experiments demonstrated that Urokinase and Alteplase injections caused blood vessel rupture and subsequent bleeding in zebrafish. These injections resulted in the thickening of the blood vessel wall and accumulation of endothelial cells on the wall, suggesting potential damage to the vascular structure. In contrast, zebrafish injected with rhTNK exhibited only minimal bleeding, without significant damage to the vascular wall or vascular endothelial cells. This indicates that the rhTNK injection had a lesser propensity to cause bleeding and vascular damage compared to Urokinase and Alteplase in this experimental model. These results suggest that the third-generation thrombolytic drug, rhTNK, shows a relatively safe efficacy profile in terms of minimizing the bleeding and preserving the vascular integrity compared to older generation thrombolytic agents like Urokinase and Alteplase. The results corroborate the idea that the progress in the development of thrombolytic drugs has resulted in enhanced safety profiles, underscoring the significance of the ongoing research and innovation in this domain.

The verification of the coagulation and clotting-related genes provided additional insights into the effects of Urokinase, Alteplase, and rhTNK on the gene expression associated with the clotting mechanisms. The study revealed that Urokinase had the most significant inhibitory effect on the expression of the clotting-related genes. This finding aligns with the observed outcome of Urokinase causing the substantial bleeding in previous experiments. The strong inhibition of the clotting-related gene expression by Urokinase further supports its association with the increased risk of bleeding. Following Urokinase, Alteplase also demonstrated an inhibitory effect on the clotting-related gene expression, albeit to a lesser extent. This corresponds with the known tendency of Alteplase to potentially induce bleeding events, as indicated by previous studies and the experimental results. In contrast, the injection of recombinant tissue plasminogen activator (rhTNK) did not alter the expression levels of the clotting-related genes and did not exhibit any inhibitory effects on these genes. This suggests that the rhTNK injection did not promote changes in the clotting gene expression patterns and did not inhibit the normal clotting process. This finding supports the notion that rhTNK administration is less likely to cause bleeding events compared to Urokinase and Alteplase. Overall, the results from the analysis of the clotting-related gene expression further support the relative safety profile of rhTNK compared to Urokinase and Alteplase in terms of minimizing the risk of bleeding associated with thrombolytic treatment.

## 5. Conclusions

In conclusion, the study comparing the effects of Urokinase, Alteplase, and rhTNK on cardiomyocytes, zebrafish vascular integrity, and the clotting-related gene expression provides valuable insights into the safety and efficacy profiles of these thrombolytic agents. The Urokinase treatment increased the cell viability but led to structural damage in human cardiomyocytes. The Alteplase treatment reduced the cell viability and caused damage to the myocardial cytoskeleton fibers. The rhTNK treatment preserved the cell viability and structural integrity of human cardiomyocytes without adverse effects. The Urokinase and Alteplase injections resulted in blood vessel rupture, bleeding, and damage to the vascular wall in zebrafish. In contrast, the rhTNK injection caused minimal bleeding and did not damage the vascular wall or endothelial cells in zebrafish. Urokinase significantly inhibited the expression of the clotting-related genes, consistent with its propensity to cause bleeding. Alteplase also showed inhibitory effects on the clotting gene expression, though to a lesser extent. rhTNK did not alter the clotting gene expression levels, indicating a lack of inhibitory effects on the clotting mechanisms. Urokinase and Alteplase exhibited higher risks of bleeding and vascular damage compared to rhTNK. rhTNK demonstrated a relatively safe efficacy profile with a minimal impact on the clotting mechanisms and vascular integrity. rhTNK has a lower cost compared to Alteplase, along with a simpler administration method. Overall, the findings suggest that rhTNK may offer a safer alternative to Urokinase and Alteplase in thrombolytic therapy, with potentially lower risks of bleeding and adverse effects on cardiomyocytes and vascular structures. Further research and clinical studies are warranted to validate these observations and optimize the use of thrombolytic agents in clinical settings. This study is the first to systematically evaluate the thrombolytic effects and side effects of three thrombolytic drugs in vivo and in vitro, and provides a strong reference for the selection of thrombolytic drugs. In future experiments involving human subjects, it would be important to assess the safety and efficacy of rhTNK in comparison to Urokinase and Alteplase. Clinical trials can be conducted to evaluate the thrombolytic effects, bleeding risks, and adverse events associated with these drugs. Large-scale randomized controlled trials can provide robust evidence on the clinical outcomes and long-term effects of these thrombolytic agents.

## Data Availability

The original contributions presented in the study are included in the article; further inquiries can be directed to the corresponding author.

## Supplementary Materials

The following supporting information can be downloaded at: https://www.mdpi.com/article/10.3390/toxics12070458/s1, Figure S1: AC16 cell compared the safety of different thrombolytic drugs for 48 h; Figure S2: Effect of three thrombolytic drugs on coagulation pathway; Table S1: Sequences of primers used for qPCR.
