# Supplementary material for: Comparative Analysis of Therapeutic Efficacy and Adverse Reactions among Various Thrombolytic Agents
Source: Toxics. 2024 Jun 25;12(7):458. doi: 10.3390/toxics12070458 (PMC11280831; doi:10.3390/toxics12070458)
Supplement: Supplementary file 1 [file toxics-12-00458-s001.zip › toxics-3020642-supplementary.pdf]

Supplementary Materials

Comparative Analysis of Therapeutic Efficacy and Adverse Reactions among Various Thrombolytic Agents

Chenxi Xie [1,†], Naying Zheng [2,†], Mingmei Li [1], Zhiyang Zhang [1], Dongqin Huang [1], Meizhu Xiao [1], Dongdong Chen [1], Chengyong He [2], Zhenghong Zuo [2,*] and Xintan Chen [1,*]

[1] Chest Pain Center, Anxi County Hospital, Quanzhou 362300, China;kudistar@163.com (C.X.); 15259794157@163.com (M.L.); 15106084165@163.com (Z.Z.); huangdongqin610@163.com (D.H.); 18960200260@163.com (M.X.); chendongdong244@163.com (D.C.)

[2] State Key Laboratory of Cellular Stress Biology, School of Life Sciences, Xiamen University, Xiamen 361005, China; 18750249346@163.com (N.Z.); hecy@xmu.edu.cn (C.H.)

* Correspondence: zuozhenghong@xmu.edu.cn (Z.Z.); axxyykjk@163.com (X.C.)

† These authors contributed equally to this work.

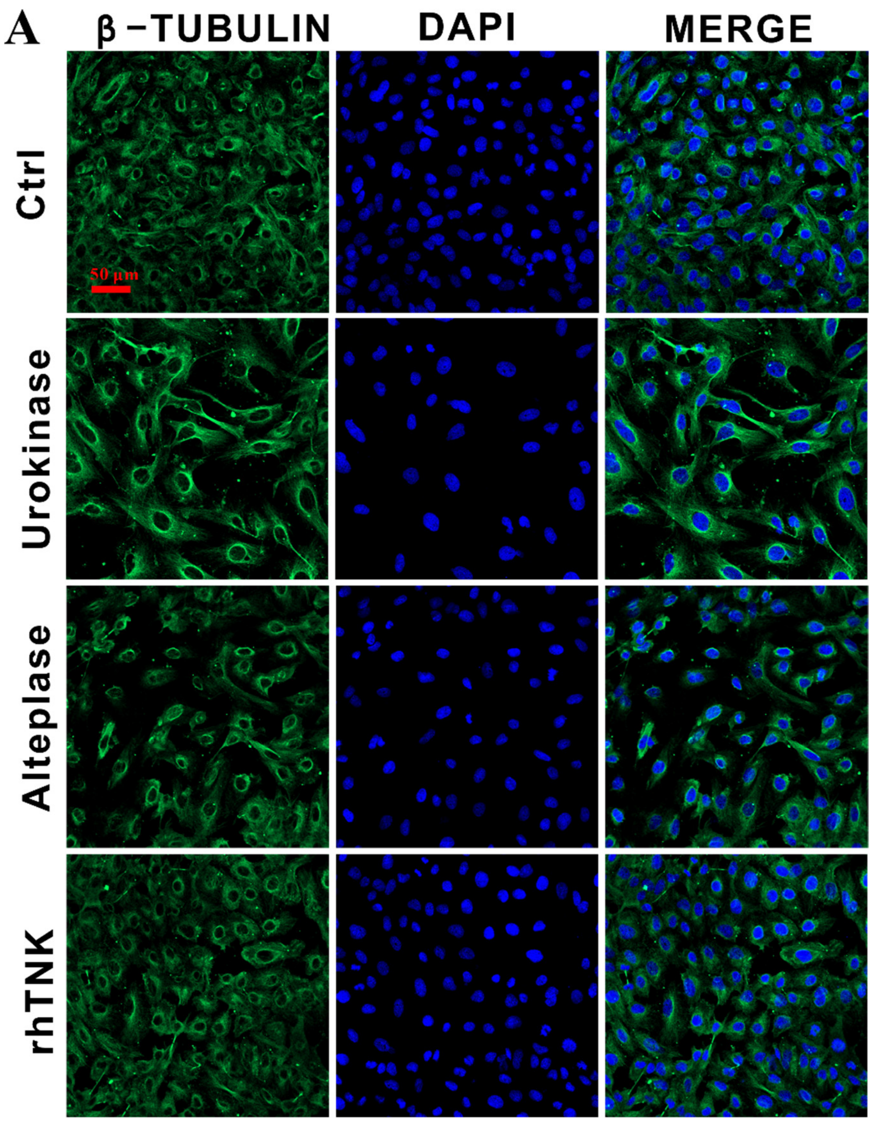

Figure S1. AC16 cell compared the safety of different thrombolytic drugs for 48 h. Cytoskeleton stained by β-TUBULIN and nuclear stained DAPI of AC 16 cells after the treatment of the three drugs, green: Cytoskeleton, blue: nuclear A).

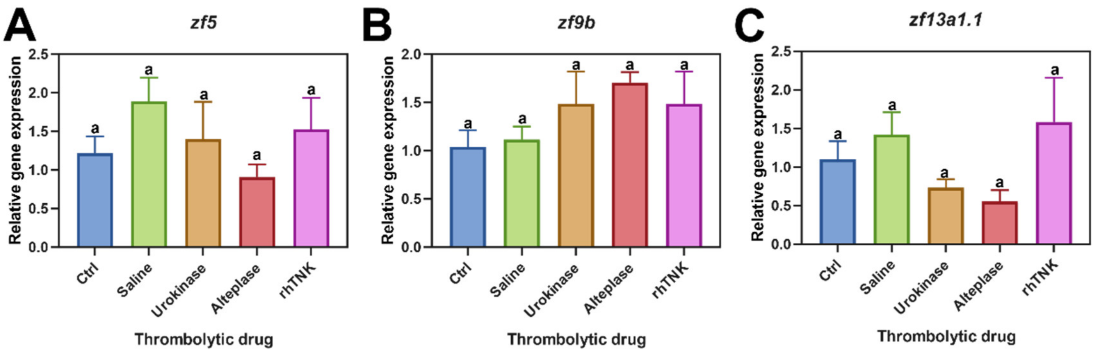

Figure S2. Effect of three thrombolytic drugs on coagulation pathway. The relative expression level of clotting factor was changed by three thrombolytic drugs, *zf5* A), *zf9b* B), *zf13a1.1* C).

Table S1 Sequences of primers used for qPCR.

Gene	Sequence	GenBank number
zf11r1.1	F:5'-TCTCACTCTTCGTCACAGGC-3'<br>R:5'-TCCACCACTTCCAGACACCT-3'	NM_001004667.3
zf11r1.2	F:5'CAGTTTTGTGCGGACCTAGC-3'<br>R:5'-CCAAAGTCAGCGGTGTAGG-3'	NM_001082982.1
zf13a1.1	F:5'-GTCACCAAAAGGCCCAACAC-3'<br>R:5'-GAGACTGACGGTGCTGGTTT-3'	NM_001076711.2
zf9a	F:5'-CGAACACATGGCTGTTTGAGG-3'<br>R:5'-ACAAAACCTGGCAGACAGTTG-3'	NM_182868.2
zf13a1b	F:5'-CCAGCTGATCCGGTGTTTCT-3'<br>R:5'-AGTTTCCCACCAACACTCCG-3'	XM_021477561.1
zf9b	F:5'-TGCCGAGGGTTACAGATTGG-3'<br>R:5'-TTTCGAGGTGTGCTTGTGCT-3'	NM_001040310.1
zf5	F:5'-CATAGGAGTGCCAAGACGGG-3'<br>R:5'-TTATCAAGTCGCCTCTGCCC-3'	NM_001328531.1
zf7	F:5'-AGTCACGAGGTGTTTCCGTG-3'<br>R:5'-GCTCACCTGCCACTATCCTG-3'	NM_131819.2
zf8	F:5'-TGTGGAGCGTTGGGAAAACT-3'<br>R:5'-TCTAACCCGACAACGCTTCC-3'	XM_009307649.3
zserpinc1	F:5'-ACACCGGGCAAGATGAAGTTA-3'<br>F:5'-TGAAAAGCGAGAGGGCGAA-3'	NM_182863.1
β-actin	F: 5'-AAGCAGGAGTACGATGAGTC-3'<br>R: 5'-TGGAGTCCTCAGATGCATTG-3'	BC045846.1

F: forward primer; R: reverse primer.
